# Supplementary material for: Identity-by-descent segments in large samples
Source: bioRxiv. 2025 Jan 7:2024.06.05.597656. Originally published 2024 Jun 8. Preprint. [Version 2] doi: 10.1101/2024.06.05.597656 (PMC11185678; doi:10.1101/2024.06.05.597656)
Supplement: 1 [file NIHPP2024.06.05.597656V2-supplement-1.pdf]

bioRxiv preprint doi: https://doi.org/10.1101/2024.06.05.597656; this version posted January 7, 2025. The copyright holder for this preprint (which was not certified by peer review) is the author/funder, who has granted bioRxiv a license to display the preprint in perpetuity. It is made available under aCC-BY 4.0 International license.

A.1. Derivations of theoretical results

A.1.1. Theorem 3.1 and its extensions

Lemma A.1. $\mathbb{E}_2[X_{a,b}] \to 0$ *uniformly as* $Nw \to \infty$.

Proof. Let $f(N) = (Nw)^{-1}$, and recall that $E_2[X_{a,b}] = (2Nw+1)^{-1}$. If $Nw > (1/\varepsilon - 1)/2$, then $|f(N) - 0| < \varepsilon$. Choose integer M such that $Mw \geq (1/\varepsilon - 1)/2$. Thus, for $\varepsilon > 0$, there exists M such that $|f(N)| = (2Nw+1)^{-1} < \varepsilon$ for all $N \geq M$. □

Lemma A.2. *Let* $X \sim$ *Bernoulli*(q) *and* $q \in (0,1)$. $\mathbb{E}[|Z|^3]/\mathbb{E}[|Z|^2]^{3/2}$ *is bounded above where* $Z = X - \mathbb{E}[X]$.

Proof.

$$\begin{aligned}\mathbb{E}[|Z|^3] &= |1-q|^3 q + |q|^3(1-q) \\ &= q(1-q)((1-q)^2 + q^2) \\ &< 1.\end{aligned} \tag{A.1}$$

$$\begin{aligned}\mathbb{E}[|Z|^2]^{3/2} &= (|1-q|^2 q + |q|^2(1-q))^{3/2} \\ &= (q(1-q)(1-q+q))^{3/2} \\ &= (q(1-q))^{3/2} \\ &> 0.\end{aligned} \tag{A.2}$$

□

Lemma A.3. *Cov*$_3(Z_{a,b}, Z_{a,c}) \equiv$ *Cov*$_3(X_{a,b}, X_{a,c}) = O((Nw)^{-2})$.

Proof. Up to reordering three sample haplotypes, there is one possible bifurcating tree (Figure S9). Sample haplotypes a and b coalesce to a common ancestor, and

bioRxiv preprint doi: https://doi.org/10.1101/2024.06.05.597656; this version posted January 7, 2025. The copyright holder for this preprint (which was not certified by peer review) is the author/funder, who has granted bioRxiv a license to display the preprint in perpetuity. It is made available under aCC-BY 4.0 International license.

their common ancestor coalesces to a common ancestor with sample haplotype c. We integrate over coalescent time and haplotype segment lengths to bound the covariance.

$$\begin{aligned}\mathbb{E}_3[X_{a,b}] &= 3\int \exp(-2Nt_3w)\exp(-3t_3)dt_3 \\ &= 3(2Nw+3)^{-1}.\end{aligned} \tag{A.3}$$

$$\begin{aligned}\mathbb{E}_3[X_{a,c}] &= 3\int\int \exp(-2Nt_3w)\exp(-2Nt_2w)\exp(-3t_3))\exp(-t_2)dt_3dt_2 \\ &= 3(2Nw+1)^{-1}(2Nw+3)^{-1}.\end{aligned} \tag{A.4}$$

$$\begin{aligned}\mathbb{E}_3[X_{a,b}X_{a,c}] &= 3\int\int\int \exp(-3Nt_3w)\exp(-2Nt_2w)\exp(-3t_3)\exp(-t_2)dt_3dt_2 \\ &= (2Nw+1)^{-1}(Nw+1)^{-1}.\end{aligned} \tag{A.5}$$

$$\begin{aligned}\mathrm{Cov}_3(X_{a,b},X_{a,c}) &= \mathbb{E}_3[X_{a,b}X_{a,c}] - \mathbb{E}_3[X_{a,b}]\cdot\mathbb{E}_3[X_{a,c}] \\ &= (2Nw+1)^{-1}((Nw+1)^{-1} - 9(2Nw+1)^{-1}(2Nw+3)^{-1}) \\ &\leq (2Nw+1)^{-1}(Nw)^{-1} \\ &= O((Nw)^{-2}).\end{aligned} \tag{A.6}$$

□

Lemma A.4. *$Cov_4(Z_{a,b}, Z_{c,d}) \equiv Cov_4(X_{a,b}, X_{c,d}) = O((Nw)^{-3})$.*

Proof. Up to reordering four sample haplotypes, there are two possible bifurcating trees (Figure S10). The first tree is as follows: sample haplotypes a and b coalesce to a common ancestor, then sample haplotypes c and d coalesce to a common

bioRxiv preprint doi: https://doi.org/10.1101/2024.06.05.597656; this version posted January 7, 2025. The copyright holder for this preprint (which was not certified by peer review) is the author/funder, who has granted bioRxiv a license to display the preprint in perpetuity. It is made available under aCC-BY 4.0 International license.

ancestor, and finally those common ancestors coalesce. The covariance of $X_{a,b}$ and $X_{c,d}$ is zero because of independent meioses. We focus instead on the covariance of $X_{a,c}$ and $X_{b,d}$. We integrate over coalescent time and haplotype segment lengths to bound the covariance.

$$\begin{aligned}\mathbb{E}_4[X_{a,c}] &= \mathbb{E}_4[X_{b,d}] \\ &= 6 \cdot 3 \int\int \exp(-2N(t_4+t_3+t_2)w)\exp(-(6t_4+3t_3+t_2))\, dt_4 dt_3 dt_2 \\ &= 18(2Nw+6)^{-1}(2Nw+3)^{-1}(2Nw+1)^{-1}.\end{aligned} \tag{A.7}$$

$$\begin{aligned}\mathbb{E}_4[X_{a,c}X_{b,d}] = 6 \cdot 3 \int\int\int &\exp(-(4Nt_4+3Nt_3+2Nt_2)w) \\ &\exp(-(6t_4+3t_3+t_2))dt_4dt_3dt_2 \\ = 18(4Nw&+6)^{-1}(3Nw+3)^{-1}(2Nw+1)^{-1}.\end{aligned} \tag{A.8}$$

$$\mathrm{Cov}_4(X_{a,c}, X_{b,d}) \leq 3(4Nw+6)^{-1}(Nw+1)^{-1}(2Nw+1)^{-1} = O((Nw)^{-3}). \tag{A.9}$$

The second tree is as follows: a and b coalesce to a common ancestor, then their common ancestor coalesces with c, and finally, the common ancestor of a, b, and c coalesces with d. It is easy to verify that $\mathbb{E}_4[X_{a,c}X_{b,d}]$ is the exact same as in Equation A.8. Next,

$$\begin{aligned}\mathbb{E}_4[X_{a,c}] &= 6 \cdot 3 \int\int \exp(-2N(t_4+t_3)w)\exp(-(6t_4+3t_3+t_2))\, dt_4 dt_3 dt_2 \\ &= 18(2Nw+6)^{-1}(2Nw+3)^{-1}.\end{aligned} \tag{A.10}$$

bioRxiv preprint doi: https://doi.org/10.1101/2024.06.05.597656; this version posted January 7, 2025. The copyright holder for this preprint (which was not certified by peer review) is the author/funder, who has granted bioRxiv a license to display the preprint in perpetuity. It is made available under aCC-BY 4.0 International license.

$$\begin{aligned}\mathbb{E}_4[X_{b,d}] &= 6 \cdot 3 \int\int \exp(-2N(t_4+t_3+t_2)w)\exp(-(6t_4+3t_3+t_2))\, dt_4 dt_3 dt_2 \\ &= 18(2Nw+6)^{-1}(2Nw+3)^{-1}(2Nw+1)^{-1}.\end{aligned} \tag{A.11}$$

Because Equations A.10 and A.11 are nonnegative, the marginal covariance upper bound is the same as in Equation A.9. □

Lemma A.5. *The following are true*

- $Cov_2(\tilde{Z}_{a,b}, \tilde{Z}_{a,b}) \equiv Cov_2(Y_{a,b}, Y_{a,b}) = O((Nw)^{-1})$;
- $Cov_3(\tilde{Z}_{a,b}, \tilde{Z}_{a,c}) \equiv Cov_3(Y_{a,b}, Y_{a,c}) = O((Nw)^{-2})$;
- $Cov_4(\tilde{Z}_{a,c}, \tilde{Z}_{b,d}) \equiv Cov_4(Y_{a,c}, Y_{b,d}) = O((Nw)^{-3})$.

Proof. We take the same approach as in Lemmas A.3 and A.4, except the survival function is that of an Erlang random variable with shape parameter 2.

$$\begin{aligned}\mathbb{E}_2[Y_{a,b}] &= \int (\exp(-2Nt_2w) + 2Nt_2w\exp(-2Nt_2w))\exp(-t_2)dt_2 \\ &= (2Nw+1)^{-1} + \int 2Nt_2w\exp(-(2Nw+1)t_2)dt_2 \\ &= (2Nw+1)^{-1} + 2Nw\int t_2\exp(-(2Nw+1)t_2)dt_2 \\ &= (2Nw+1)^{-1} + 2Nw(2Nw+1)^{-2} \\ &= (2Nw+1)^{-1}(1+2Nw(2Nw+1)^{-1}).\end{aligned} \tag{A.12}$$

bioRxiv preprint doi: https://doi.org/10.1101/2024.06.05.597656; this version posted January 7, 2025. The copyright holder for this preprint (which was not certified by peer review) is the author/funder, who has granted bioRxiv a license to display the preprint in perpetuity. It is made available under aCC-BY 4.0 International license.

$$
\begin{aligned}
\mathbb{E}_3[Y_{a,b}] &= 3\int (\exp(-2Nt_3w) + 2Nt_3w\exp(-2Nt_3w))\exp(-3t_3)dt_2 \\
&= 3((2Nw+3)^{-1} + 2Nw\int t_3\exp(-(2Nw+3)t_3)) \\
&= 3((2Nw+3)^{-1} + 2Nw(2Nw+3)^{-2}) \\
&= 3(2Nw+3)^{-1}(1 + 2Nw(2Nw+3)^{-1}).
\end{aligned}
\tag{A.13}
$$

$$
\begin{aligned}
\mathbb{E}_3[Y_{a,c}] &= 3(2Nw+3)^{-1}(2Nw+1)^{-1} \\
&\quad + 6Nw\int (t_3+t_2)\exp(-(2Nw+3)t_3)\exp(-(2Nw+1)t_2)dt_3dt_2 \\
&= 3((2Nw+3)^{-1}(2Nw+1)^{-1} + 2Nw(2Nw+3)^{-2}(2Nw+3)^{-2}) \\
&= 3(2Nw+3)^{-1}(2Nw+1)^{-1}(1 + 2Nw(2Nw+3)^{-1}(2Nw+3)^{-1}).
\end{aligned}
\tag{A.14}
$$

From Equations A.12, A.13, and A.14, the pattern emerges that the effect of the convolution of crossover points is to multiply $O(1)$ terms to the marginal expected values in Equation 4 and Lemmas A.3 and A.4.

Calculating $\mathbb{E}_3[Y_{a,b}Y_{a,c}]$ is more involved. Up to reordering three sample haplotypes, we consider sample haplotypes a and c that coalesce at the most recent common ancestor of a, b, and c. Then, $\mathbb{E}_3[Y_{a,c}] \geq \mathbb{E}_3[Y_{a,b}Y_{a,c}]$, and

$$
\begin{aligned}
\mathrm{Cov}_3(Y_{a,b}, Y_{a,c}) &= \mathbb{E}_3[Y_{a,b}Y_{a,c}] - \mathbb{E}_3[Y_{a,b}]\mathbb{E}_3[Y_{a,c}] \\
&\leq \mathbb{E}_3[Y_{a,b}Y_{a,c}] \\
&\leq \mathbb{E}_3[Y_{a,c}] \\
&= O((Nw)^{-2}).
\end{aligned}
\tag{A.15}
$$

Using the same techniques, it is easy to calculate $\mathbb{E}_4[Y_{a,c}]$ and $\mathbb{E}_4[Y_{b,d}]$ for the

bioRxiv preprint doi: https://doi.org/10.1101/2024.06.05.597656; this version posted January 7, 2025. The copyright holder for this preprint (which was not certified by peer review) is the author/funder, who has granted bioRxiv a license to display the preprint in perpetuity. It is made available under aCC-BY 4.0 International license.

two different tree shapes and derive the $O((Nw)^{-3})$ bound for $\text{Cov}_4(Y_{a,c}, Y_{b,d})$.

$\square$

Lemma A.6. *For a sample of three haplotypes a, b, and c, when $\mathbb{E}_2[X_{a,c}] < 1/2$, the conditional expectation $\mathbb{E}[Z_{a,c} \times \mathbf{Z}_{-a,c}|\mathbf{Z}_{-a,c}] \not\geq 0$ for all $\mathbf{Z}_{-a,c}$.*

Proof. Define $q =: \mathbb{E}_2[X_{a,c}]$, and fix $\mathbf{X}_{-a,c} = 1$.

$$\begin{aligned}\mathbb{E}[Z_{a,c} \times \mathbf{Z}_{-a,c}|\mathbf{Z}_{-a,c}] &= \mathbb{E}[(X_{a,c} - q) \times (X_{a,b} + X_{b,c} - 2q)|X_{a,b} + X_{b,c} = 1] \\ &= \mathbb{E}[X_{a,c} \times (1 - 2q)|X_{a,b} + X_{a,c} = 1] - q + 2q^2\end{aligned}$$

Because of IBD transitivity, $X_{a,c} = 0$ with probability 1. Then, the equation simplifies to $-q(1 - 2q) < 0$. $\square$

A.1.2. Multi-way IBD segments

Proof of Theorem 4.2. We give the general argument for 3-way IBD segment indicators. To begin, we calculate bounds on the relevant integrals $\mathbb{E}_k[\cdot], \ldots, \mathbb{E}_{2k}[\cdot]$. Recall that $\mathbb{E}_k$ is the expected value with respect to a coalescent tree of k haplotypes.

$$\begin{aligned}\mathbb{E}_3[X_{a,b,c}X_{a,b,c}] &= O((Nw)^{-2}) \\ \mathbb{E}_4[X_{a,b,c}X_{a,b,d}] &= O((Nw)^{-3}) \\ \mathbb{E}_5[X_{a,b,c}X_{a,d,e}] &= O((Nw)^{-4}) \\ \mathbb{E}_6[X_{a,b,c}X_{d,e,f}] &= O((Nw)^{-5}).\end{aligned} \quad \text{(A.16)}$$

These are also the covariance bounds because $\mathbb{E}_3[X_{a,b,c}X_{a,b,c}] \geq 0$ and $\mathbb{E}_3[X_{a,b,c}X_{a,b,c}] \geq \mathbb{E}_3[X_{a,b,c}]^2$ and so on for the other $\mathbb{E}_k$ relations.

bioRxiv preprint doi: https://doi.org/10.1101/2024.06.05.597656; this version posted January 7, 2025. The copyright holder for this preprint (which was not certified by peer review) is the author/funder, who has granted bioRxiv a license to display the preprint in perpetuity. It is made available under aCC-BY 4.0 International license.

Next, we take sums over these covariance bounds and substitute in the $n = o(Nw)$ condition.

$$\begin{aligned}\Omega_{\binom{n}{3}} &\sim n^3 \cdot O((Nw)^{-2}) \\ &= o((Nw)^3) \cdot O((Nw)^{-1}) \\ &= o(Nw);\end{aligned} \tag{A.17}$$

$$\begin{aligned}\sum_{a,b,c,d} \mathrm{Cov}_4(X_{a,b,c}, X_{a,b,d}) &\sim n^4 \cdot O((Nw)^{-3}) \\ &= o((Nw)^4) \cdot O((Nw)^{-3}) \\ &= o(Nw);\end{aligned} \tag{A.18}$$

$$\begin{aligned}\sum_{a,b,c,d,e} \mathrm{Cov}_5(X_{a,b,c}, X_{a,d,e}) &\sim n^5 \cdot O((Nw)^{-4}) \\ &= o((Nw)^5) \cdot O((Nw)^{-4}) \\ &= o(Nw);\end{aligned} \tag{A.19}$$

$$\begin{aligned}\sum_{a,b,c,d,e,f} \mathrm{Cov}_6(X_{a,b,c}, X_{d,e,f}) &\sim n^6 \cdot O((Nw)^{-5}) \\ &= o((Nw)^6) \cdot O((Nw)^{-5}) \\ &= o(Nw).\end{aligned} \tag{A.20}$$

The covariance within IBD segment indicators $\Omega_{\binom{n}{3}}$ controls the sum of covariances $\sum_{(a,b,c)\neq(d,e,f)} \mathrm{Cov}(X_{a,b,c}, X_{d,e,f})$. Using the bounding argument in Equation A.15, the result extends to IBD segment indicators around a focal location.

□

bioRxiv preprint doi: https://doi.org/10.1101/2024.06.05.597656; this version posted January 7, 2025. The copyright holder for this preprint (which was not certified by peer review) is the author/funder, who has granted bioRxiv a license to display the preprint in perpetuity. It is made available under aCC-BY 4.0 International license.

A.1.3. Multivariate IBD rates

Proof of Theorem 4.3. To use Corollary 1 from Chandrasekhar et al. [12] in multiple dimensions, we now require defining their notion of an affinity set. These are subsets $\mathcal{A}^l_{a,b}$ containing the haplotype pair a and b from sample l such that $\text{Cov}(X^l_{a,b}, X^{l^\star}_{c,d})$ is high if the haplotype pair c and d from sample $l^\star$ are in the affinity set and low if they are not. We consider the singleton affinity sets $\{X^l_{a,b}\}$. (We remark that singletons are the affinity sets we use in all of our proofs for the one-dimensional results.)

We use the example of two sample means to concretely calculate covariances. Let $\Omega_{2\times 2}$ be the covariance matrix such that

$$\Omega_{0,0} = \Omega_{1,1} = \sum \text{Cov}(X^0_{a,b}, X^0_{a,b}) \sim n^2(Nw)^{-1};$$
$$\Omega_{0,1} = \Omega_{1,0} = 0.$$

$\Omega_{0,1}$ and $\Omega_{1,0}$ concern the sum of covariances of IBD segment indicators within affinity sets but in different samples, which is zero because the affinity set of a haplotype pair in one sample includes no haplotype pairs in a different sample.

The term that controls the sum of covariances across affinity sets is the Frobenius norm $||\Omega_{2\times 2}||_F$. We calculate this norm as

$$\begin{aligned} ||\Omega_{2\times 2}||_F &= \sqrt{\Omega^2_{0,0} + 2\cdot\Omega^2_{0,1} + \Omega^2_{1,1}} \\ &\sim \sqrt{2n^4(Nw)^{-2} + 0} \\ &= \sqrt{2}n^2(Nw)^{-1}. \end{aligned} \tag{A.21}$$

Under the condition $n = o(Nw)$, Equation A.21 is $o(Nw)$, and under the condition

bioRxiv preprint doi: https://doi.org/10.1101/2024.06.05.597656; this version posted January 7, 2025. The copyright holder for this preprint (which was not certified by peer review) is the author/funder, who has granted bioRxiv a license to display the preprint in perpetuity. It is made available under aCC-BY 4.0 International license.

$Nw = o(n^2)$, the variance term $||\Omega_{2\times 2}||_F$ tends to infinity.

The first condition from Corollary 1 in Chandrasekhar et al. [12] is

$$\sum_{(l^\star,a,b)\neq(l,c,d)} \mathrm{Cov}(X^{l^\star}_{a,b}, X^{l}_{c,d}) = o(||\Omega_{2\times 2}||_F) = o(Nw). \tag{A.22}$$

We compute the sums of covariances of IBD segment indicator types $\{(a,b),(a,e)\}$ and $\{(a,b),(c,d)\}$, where a, b, and e are haplotypes in one sample and c and d are haplotypes in the other sample. By the same calculations as in the previous proofs, these sums are asymptotically equivalent to $n^3(Nw)^{-2} = o(Nw)$ and $n^4(Nw)^{-3} = o(Nw)$.

Since the column vector is now multi-dimensional, we must also show that

$$\sum_{(l^\star,a,b),(l,c,d)} \mathrm{Cov}((X^{l^\star}_{a,b})^2, (X^{l}_{c,d})^2) = o(||\Omega_{2\times 2}||_F^2). \tag{A.23}$$

This calculation is simplified as

$$\begin{aligned}\sum_{(l^\star,a,b),(l,c,d)} \mathrm{Cov}((X^{l^\star}_{a,b})^2, (X^{l}_{c,d})^2) &= \sum_{(l^\star,a,b),(l,c,d)} \mathrm{Cov}(X^{l^\star}_{a,b}, X^{l}_{c,d}) \\ &= \Omega_{0,0} + \Omega_{1,1} + \sum_{(l^\star,a,b)\neq(l,c,d)} \mathrm{Cov}(X^{l^\star}_{a,b}, X^{l}_{c,d}) \\ &= o(Nw).\end{aligned} \tag{A.24}$$

We get the general result by extending these calculations for sums and norms over covariances of two samples to those of ℓ samples. The term in Equation A.22 involves sums of covariances of $\binom{\ell}{2}$ pairs of samples. This term is why we require the bound on $\binom{\ell}{2}$, because in Equation A.21 we have the multiplicative factor $\sqrt{\ell}$.

bioRxiv preprint doi: https://doi.org/10.1101/2024.06.05.597656; this version posted January 7, 2025. The copyright holder for this preprint (which was not certified by peer review) is the author/funder, who has granted bioRxiv a license to display the preprint in perpetuity. It is made available under aCC-BY 4.0 International license.

Using the bounding argument in Equation A.15, the result extends to IBD segment indicators around a focal location.

□

A.2. Verifying an assumption of the central limit theorem

We take a Monte Carlo approach to examine the conditional expectation assumption $\mathbb{E}[\tilde{Z}_{a,b} \times \tilde{\mathbf{Z}}_{-a,b}|\tilde{\mathbf{Z}}_{-a,b}] \geq 0$ for all $\tilde{\mathbf{Z}}_{-a,b}$ because $\mathbb{E}[\tilde{Z}_{a,b}|\tilde{\mathbf{Z}}_{-a,b}]$ is analytically intractable. Namely, by replacing the expected value $\mathbb{E}[Y_{a,b}|\mathbf{Y}_{-a,b}]$ with an average over a large number of simulations, we assess if $\mathbb{E}[Y_{a,b}|\mathbf{Y}_{-a,b}] \geq \mathbb{E}[Y_{a,b}]$ when $\mathbf{Y}_{-a,b} \geq (\binom{n}{2} - 1) \cdot \mathbb{E}[Y_{a,b}]$ and vice versa that $\mathbb{E}[Y_{a,b}|\mathbf{Y}_{-a,b}] \leq \mathbb{E}[Y_{a,b}]$ when $\mathbf{Y}_{-a,b} \leq (\binom{n}{2} - 1) \cdot \mathbb{E}[Y_{a,b}]$. (Recall that $Z_{a,b}$ is the binary random variable $Y_{a,b}$ after mean-centering.) The intuition is that if the observed sum $\mathbf{Y}_{-a,b}$ is larger than the expected sum $\mathbb{E}[\mathbf{Y}_{-a,b}]$ then the held out $Y_{a,b}$ is more likely to be 1 than it would be if the observed sum equaled the expected sum.

We run the Temple et al. [49] algorithm one hundred and twenty million times, recording the value of $Y_{a,b}$ and the sum $\mathbf{Y}_{-a,b}$ for some fixed haplotype pair a and b. Then, we calculate the difference between the empirical average $\bar{Y}_{a,b}$ and $\mathbb{E}[Y_{a,b}]$, stratified into eight quantile bins depending on the sum $\mathbf{Y}_{-a,b}$. The sample sizes are limited to two to four hundred diploid individuals to keep runtime modest.

Figure S11 shows the results of this simulation study. For each bin, the average count is less than and greater than $\mathbb{E}[Y_{a,b}]$ when the sum $\mathbf{Y}_{-a,b}$ is less than and greater than $\mathbb{E}[\mathbf{Y}_{-a,b}]$, respectively. This trend is especially apparent for $\mathbf{Y}_{-a,b}$ far from the mean IBD count $(\binom{n}{2} - 1) \times \mathbb{E}[Y_{a,b}]$. These findings provide empirical evidence that the theorem assumption may be true for moderate to large sample sizes.

bioRxiv preprint doi: https://doi.org/10.1101/2024.06.05.597656; this version posted January 7, 2025. The copyright holder for this preprint (which was not certified by peer review) is the author/funder, who has granted bioRxiv a license to display the preprint in perpetuity. It is made available under aCC-BY 4.0 International license.

926 # Supplementary figures

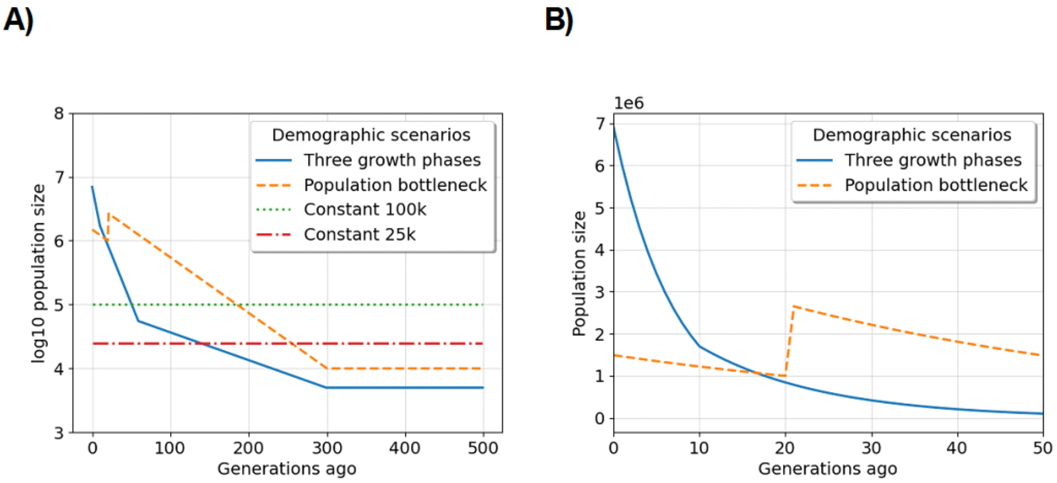

Figure S1: Demographic scenarios we consider in simulation studies: A) coalescent time in generations ago by the log 10 population size, and B) the most recent fifty generations by population size for examples of exponential growth. The legends specify the color and line style for each scenario. As opposed to coalescent time used in the main text, we describe the scenarios forward in time here. Three phases of exponential growth: a population of ancestral size five thousand diploids increases exponentially each generation at rates one, seven, and fifteen percent starting three hundred, sixty, and ten generations ago. Population bottleneck: a population of ancestral size ten thousand diploids increases exponentially each generation at a rate of two percent starting three hundred generations ago. Otherwise, the demographic scenarios we explore here are populations of constant size twenty-five and one hundred diploids.

bioRxiv preprint doi: https://doi.org/10.1101/2024.06.05.597656; this version posted January 7, 2025. The copyright holder for this preprint (which was not certified by peer review) is the author/funder, who has granted bioRxiv a license to display the preprint in perpetuity. It is made available under aCC-BY 4.0 International license.

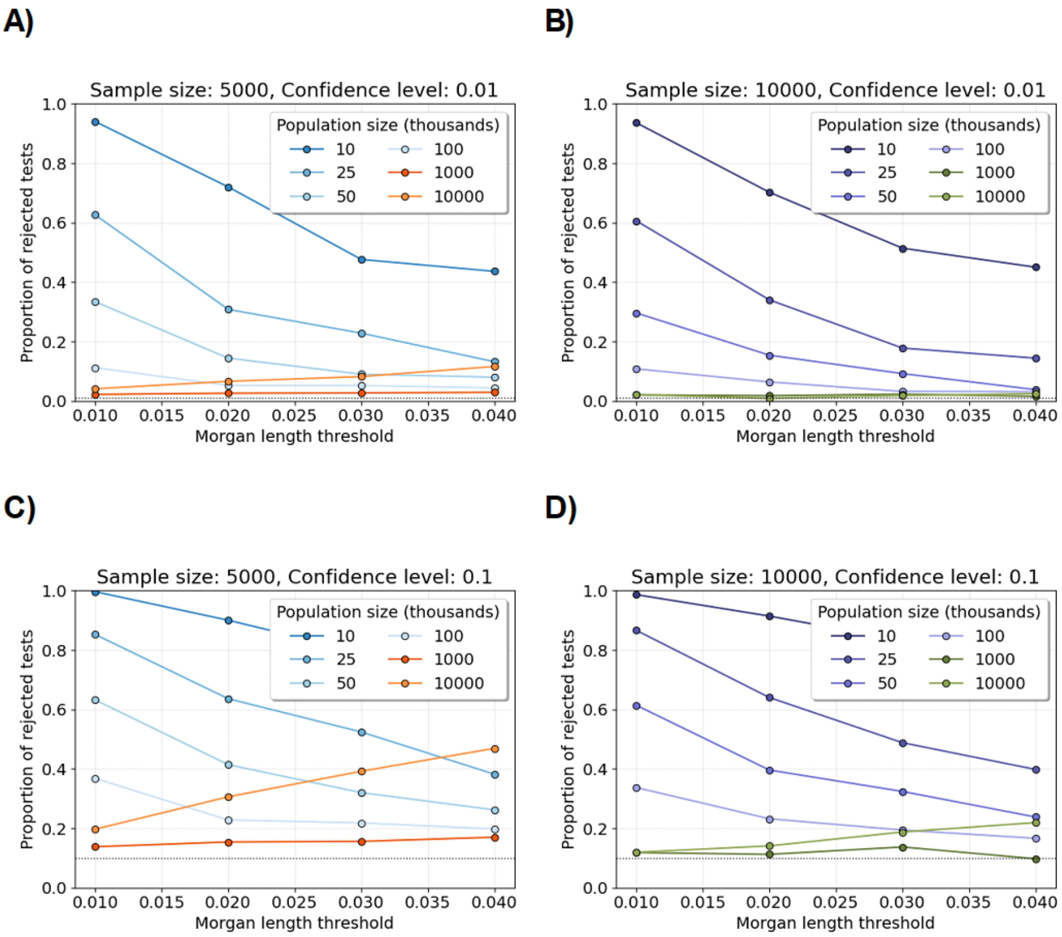

Figure S2: Shapiro-Wilk tests for varying population sizes and significance levels. Line plots show the proportions of Shapiro-Wilk tests rejected at significance levels A,B) 0.01 and C,D) 0.1 (y-axis) for varying population size and fixed sample size. Each proportion is computed over five hundred tests. Each test is based on one thousand simulations of the number of identity-by-descent lengths longer than a specified Morgans length threshold (x-axis). A,C) The sample size is five thousand diploid individuals. B,D) The sample size is ten thousand diploid individuals. The legends assign colors to different population sizes. The horizontal dotted lines are significance levels.

bioRxiv preprint doi: https://doi.org/10.1101/2024.06.05.597656; this version posted January 7, 2025. The copyright holder for this preprint (which was not certified by peer review) is the author/funder, who has granted bioRxiv a license to display the preprint in perpetuity. It is made available under aCC-BY 4.0 International license.

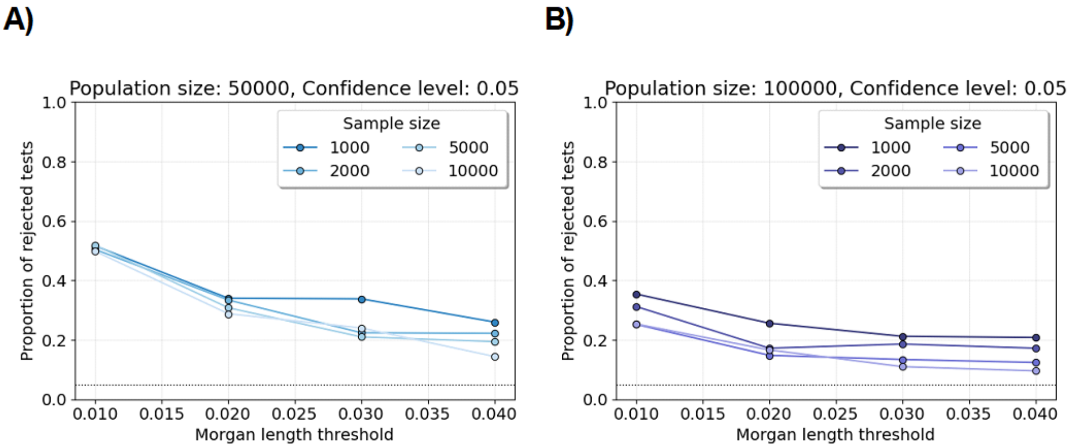

Figure S3: Shapiro-Wilk tests for varying sample sizes. Line plots show the proportions of Shapiro-Wilk tests rejected at the significance level 0.05 (y-axis) for varying sample size and fixed population size. Each proportion is computed over five hundred tests. Each test is based on one thousand simulations of the number of identity-by-descent lengths longer than a specified Morgans length threshold (x-axis). A) The population size is fifty thousand diploid individuals. B) The population size is one hundred thousand diploid individuals. The legends assign colors to different sample sizes. The horizontal dotted line is at 0.05.

bioRxiv preprint doi: https://doi.org/10.1101/2024.06.05.597656; this version posted January 7, 2025. The copyright holder for this preprint (which was not certified by peer review) is the author/funder, who has granted bioRxiv a license to display the preprint in perpetuity. It is made available under aCC-BY 4.0 International license.

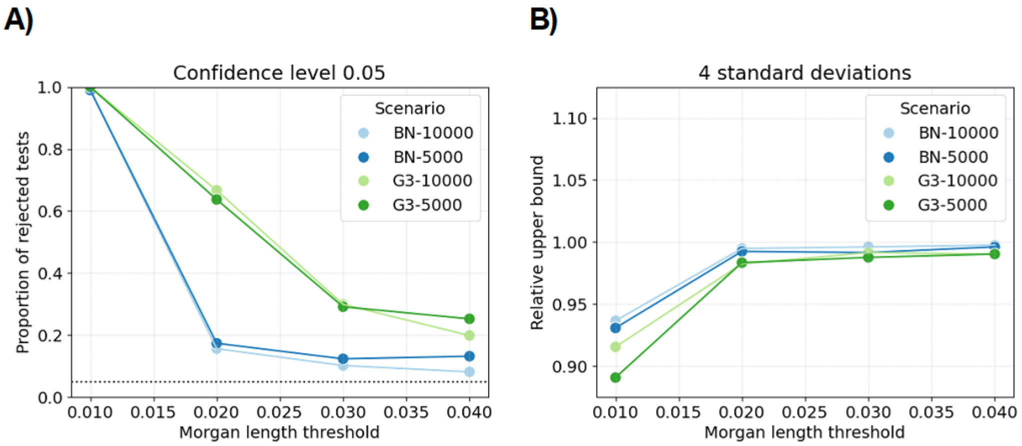

Figure S4: Shapiro-Wilk tests and relative upper tail bounds for complex demography scenarios. A) Line plots show the proportions of Shapiro-Wilk tests rejected at the sigificance level 0.05 (y-axis) for the population bottleneck (BN) or three phases of exponential growth (G3) demographic scenarios and sample sizes of five or ten thousand diploid individuals. Each proportion is computed over at least six hundred tests. Each test is based on one thousand simulations of the number of identity-by-descent lengths longer than a specified Morgans length threshold (x-axis). B) Line plots show the average mean plus four standard deviations divided by the 99.99683 percentile over two million simulations (y-axis). Plot designs are identical to Figures 2 and 3.

bioRxiv preprint doi: https://doi.org/10.1101/2024.06.05.597656; this version posted January 7, 2025. The copyright holder for this preprint (which was not certified by peer review) is the author/funder, who has granted bioRxiv a license to display the preprint in perpetuity. It is made available under aCC-BY 4.0 International license.

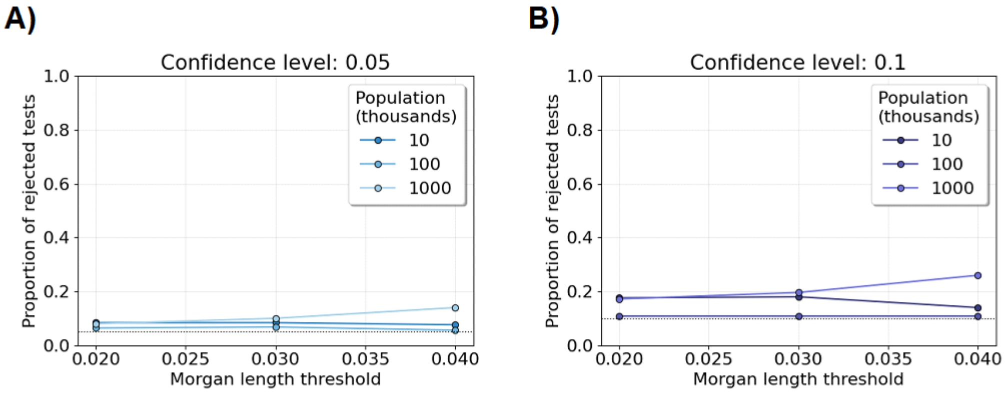

Figure S5: Shapiro-Wilk tests for difference in IBD rates between groups. Line plots show the proportions of Shapiro-Wilk tests rejected at the significance level 0.05 (y-axis) for increasing constant population sizes (in thousands). The sample size is five thousand diploid individuals. Each proportion is computed over two hundred and fifty tests. Each test is based on five hundred simulations of the difference between groups in IBD rates longer than a specified Morgans length threshold (x-axis). The significance threshold is either A) 0.05 or B) 0.10, shown as horizontal dotted black lines.

bioRxiv preprint doi: https://doi.org/10.1101/2024.06.05.597656; this version posted January 7, 2025. The copyright holder for this preprint (which was not certified by peer review) is the author/funder, who has granted bioRxiv a license to display the preprint in perpetuity. It is made available under aCC-BY 4.0 International license.

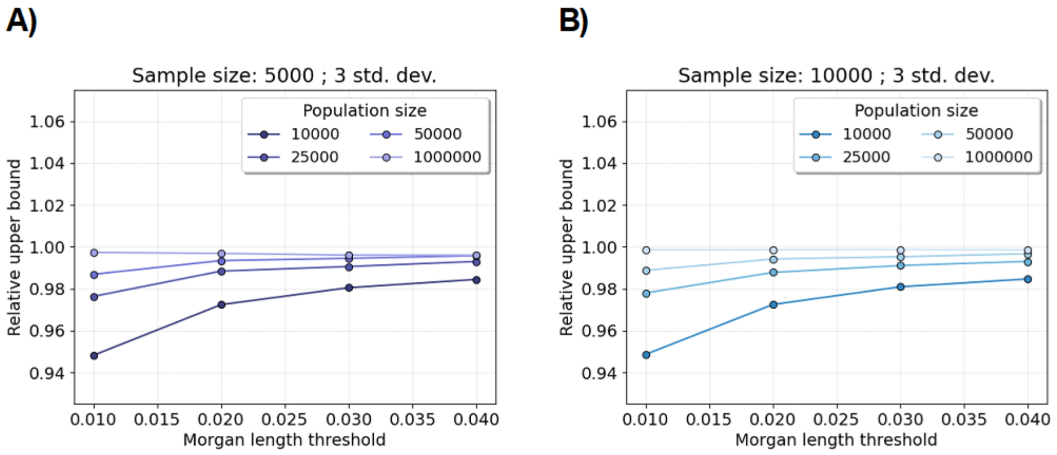

Figure S6: Relative upper bound for excess IBD scan. Line plots show the average mean plus three standard deviations divided by the 99.86501 percentile over two million simulations (y-axis). (The standard normal survival function of three is 0.9986501.) Each average relative upper bound is computed over one thousand tests. Each test is based on two thousand simulations of the number of identity-by-descent lengths longer than a specified Morgans length threshold (x-axis). A) The sample size is five thousand diploid individuals. B) The sample size is ten thousand diploid individuals. The legends assign colors to different constant population sizes.

bioRxiv preprint doi: https://doi.org/10.1101/2024.06.05.597656; this version posted January 7, 2025. The copyright holder for this preprint (which was not certified by peer review) is the author/funder, who has granted bioRxiv a license to display the preprint in perpetuity. It is made available under aCC-BY 4.0 International license.

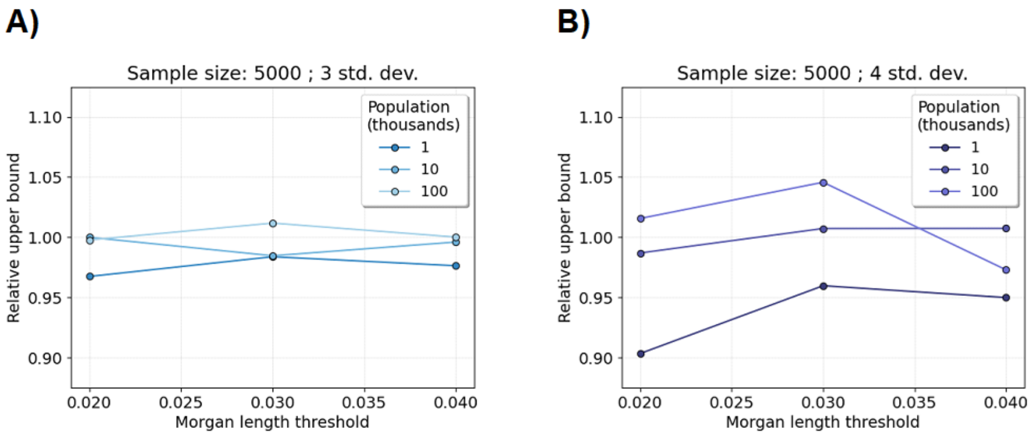

Figure S7: Relative upper bound for the difference in IBD rates test. Line plots show the average mean plus A) three or B) four standard deviations divided by the standard normal corresponding percentiles over one hundred and twenty-five thousand simulations (y-axis). Each average relative upper bound is computed over two hundred and fifty tests. Each test is based on five hundred simulations of the number of identity-by-descent lengths longer than a specified Morgans length threshold (x-axis). The sample size is five thousand diploid individuals. The legends assign colors to increasing constant population sizes (in thousands).

bioRxiv preprint doi: https://doi.org/10.1101/2024.06.05.597656; this version posted January 7, 2025. The copyright holder for this preprint (which was not certified by peer review) is the author/funder, who has granted bioRxiv a license to display the preprint in perpetuity. It is made available under aCC-BY 4.0 International license.

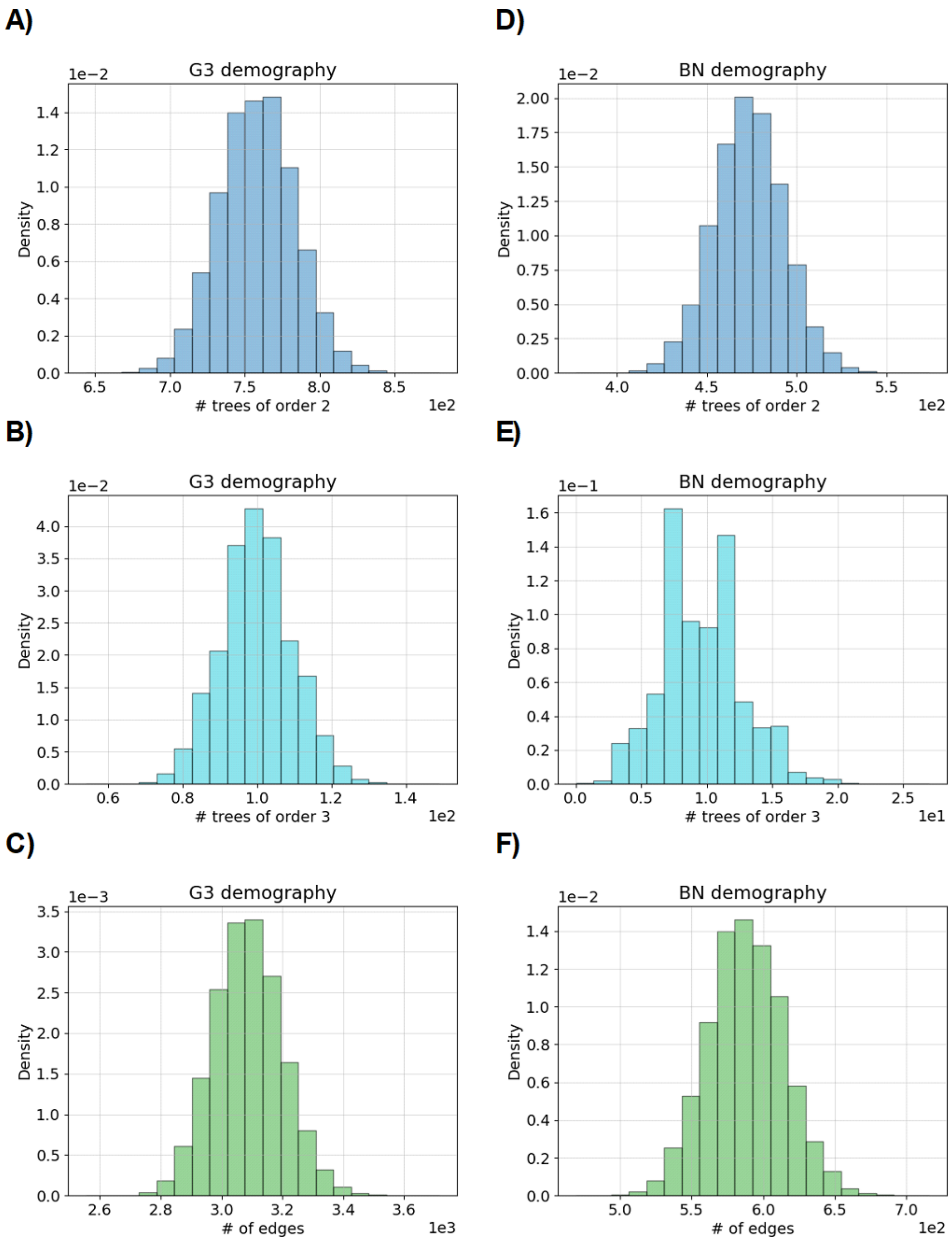

Figure S8: Comparing features between IBD graphs for complex demographic scenarios. Histograms show the density of IBD graph features between A-C) the three phases of exponential growth (G3) and D-F) the population bottleneck (BN) demographic scenarios. Each histogram is based on at least six hundred thousand simulations. A,D), B,D), and C,F) show the number of trees of order 2, the number of trees of order 3, and the total number of edges, respectively. The Morgans length threshold is 0.03. The sample size is five thousand diploid individuals.

bioRxiv preprint doi: https://doi.org/10.1101/2024.06.05.597656; this version posted January 7, 2025. The copyright holder for this preprint (which was not certified by peer review) is the author/funder, who has granted bioRxiv a license to display the preprint in perpetuity. It is made available under aCC-BY 4.0 International license.

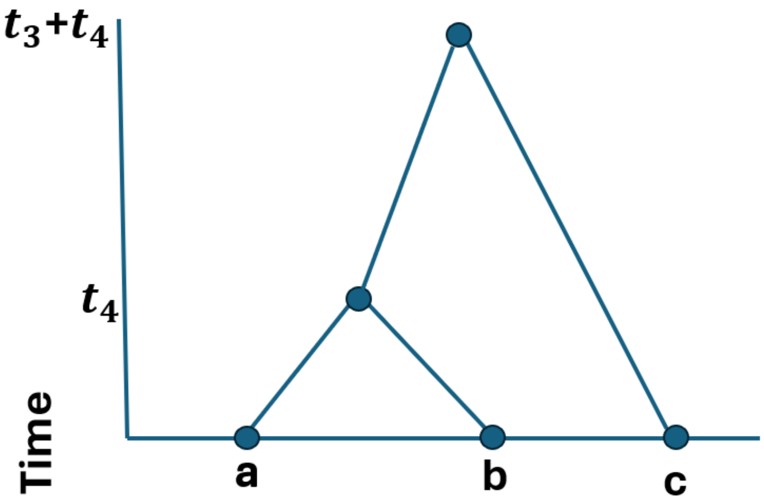

Figure S9: Illustration of the one possible coalescent tree used to calculate Cov_3 terms in Appendix A.1.

bioRxiv preprint doi: https://doi.org/10.1101/2024.06.05.597656; this version posted January 7, 2025. The copyright holder for this preprint (which was not certified by peer review) is the author/funder, who has granted bioRxiv a license to display the preprint in perpetuity. It is made available under aCC-BY 4.0 International license.

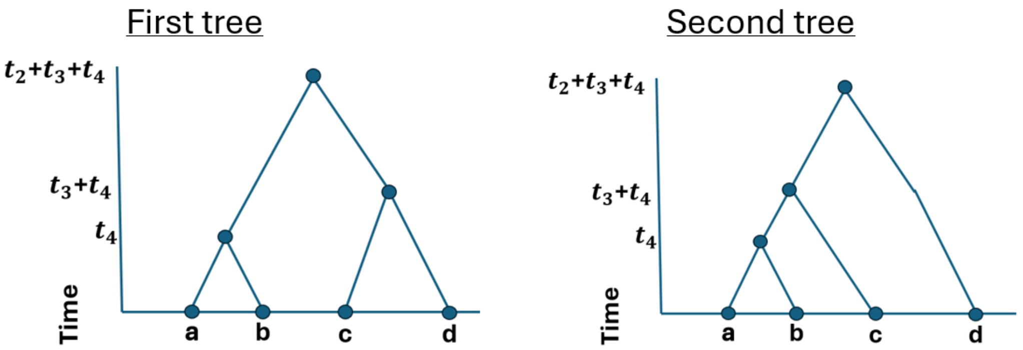

Figure S10: Illustration of the two possible coalescent trees used to calculate Cov_4 terms in Appendix A.1.

bioRxiv preprint doi: https://doi.org/10.1101/2024.06.05.597656; this version posted January 7, 2025. The copyright holder for this preprint (which was not certified by peer review) is the author/funder, who has granted bioRxiv a license to display the preprint in perpetuity. It is made available under aCC-BY 4.0 International license.

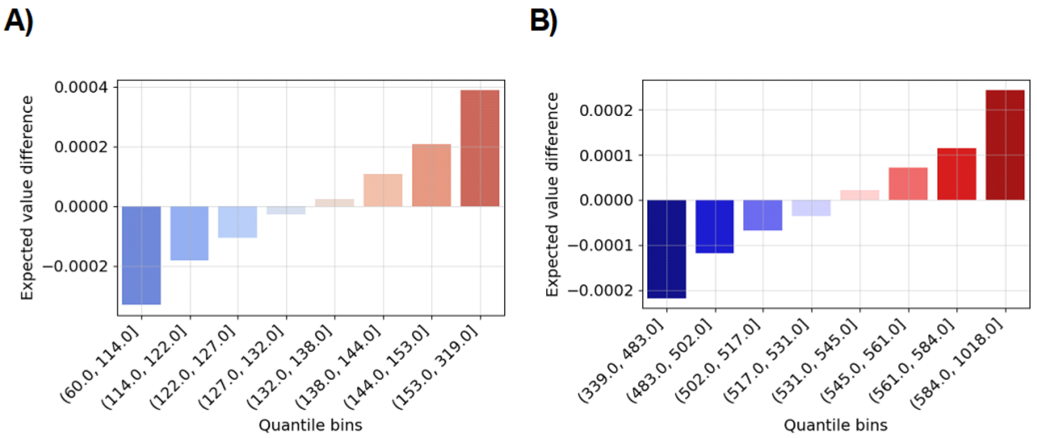

Figure S11: Monte Carlo verification of the conditional expectation condition in our central limit theorem. Bar charts show the difference between the proportion of simulations where two specific haplotypes share an IBD segment longer than 0.03 Morgans and the true success probability (y-axis). This statistic is stratified into eight quantile bins based on the total number of long IBD segments (x¬-axis). Sample sizes are A) two hundred and B) four hundred diploid individuals. The population size is ten thousand diploid individuals. The expectation is 132.78 in A) and 531.78 in B).

bioRxiv preprint doi: https://doi.org/10.1101/2024.06.05.597656; this version posted January 7, 2025. The copyright holder for this preprint (which was not certified by peer review) is the author/funder, who has granted bioRxiv a license to display the preprint in perpetuity. It is made available under aCC-BY 4.0 International license.

Supplementary tables

Type	Structure	Avg	Var	Min	Max	S.W.t.
G3	Edges	3,085.66	12,827.06	2,554.00	3,716.00	0.29
	Largest	15.60	9.22	9.00	50.00	1.00
	Tree2	757.96	635.16	644.00	880.00	0.08
	Tree3	99.71	94.29	54.00	149.00	0.42
	Complete	251.55	230.65	185.00	3,118.00	0.14
BN	Edges	587.73	694.55	469.00	716.00	0.12
	Largest	4.32	0.39	3.00	11.00	1.00
	Tree2	473.24	393.50	377.00	574.00	0.09
	Tree3	9.66	9.57	0.00	27.00	1.00
	Complete	35.53	34.76	11.00	488.00	1.00

Table S1: Summary statistics of IBD graphs for the three phases of exponential growth (G3) and the population bottleneck (BN) demographic scenarios. Network structures of interest are the number of edges (Edges), the degree of the largest components (Largest), the number of trees of order 2 and 3 (Tree-2 and Tree-3), and the number of complete components of degree 3 or more (Complete). Summary statistics are aggregated over at least six hundred thousand simulations. Shapiro-Wilk tests at the significance level 0.05 are performed with 1000 replicates for at least 600 simulations, and the proportion of rejected null hypotheses is reported as S.W.t. The sample size is five thousand diploid individuals. The Morgans length threshold is 0.03.

bioRxiv preprint doi: https://doi.org/10.1101/2024.06.05.597656; this version posted January 7, 2025. The copyright holder for this preprint (which was not certified by peer review) is the author/funder, who has granted bioRxiv a license to display the preprint in perpetuity. It is made available under aCC-BY 4.0 International license.

**Type**	**Structure**	**Avg**	**Var**	**Min**	**Max**	**S.W.t.**
$s = 0.01$	Edges	3,407.38	21,526.32	2,916.00	4,143.00	0.36
	Largest	**24.33**	50.80	11.00	89.00	0.97
	Tree2	737.77	626.12	636.00	842.00	0.05
	Tree3	95.81	92.23	57.00	138.00	0.07
	Complete	242.41	215.76	187.00	305.00	0.05
$s = 0.02$	Edges	4,693.51	140,436.48	3,579.00	8,212.00	0.95
	Largest	**73.97**	1,219.95	22.00	346.00	0.97
	Tree2	697.19	588.38	596.00	791.00	0.10
	Tree3	86.65	83.70	53.00	126.00	0.09
	Complete	220.37	199.88	161.00	281.00	0.10
$s = 0.03$	Edges	8,242.12	2,283,864.57	4,998.00	37,933.00	0.97
	Largest	**230.39**	12,224.19	39.00	819.00	0.97
	Tree2	659.10	565.21	562.00	759.00	0.07
	Tree3	78.43	74.69	46.00	119.00	0.11
	Complete	199.95	181.88	145.00	254.00	0.06
$s = 0.04$	Edges	16,486.56	24,295,227.62	7,747.00	72,775.00	0.97
	Largest	**484.92**	38,683.32	89.00	1,229.00	0.97
	Tree2	630.68	529.35	546.00	731.00	0.02
	Tree3	72.95	70.26	41.00	108.00	0.11
	Complete	185.76	167.85	135.00	241.00	0.07

Table S2: Summary statistics of IBD graphs for different selection coefficients and the three phases of exponential growth demographic scenario. There is directional selection with different selection coefficients $s \in [0.01, 0.02, 0.03, 0.4]$. The same description of IBD graph features as in Table 1. Shapiro-Wilk tests at the significance level 0.05 are performed with 250 replicates for 150 simulations, and the proportion of rejected null hypotheses reported as S.W.t. The sample size is five thousand diploid individuals. The Morgans length threshold is 0.03.

bioRxiv preprint doi: https://doi.org/10.1101/2024.06.05.597656; this version posted January 7, 2025. The copyright holder for this preprint (which was not certified by peer review) is the author/funder, who has granted bioRxiv a license to display the preprint in perpetuity. It is made available under aCC-BY 4.0 International license.

Type	Structure	Avg	Var	Min	Max	S.W.t.
$s = 0.01$	Edges	612.05	753.44	504.00	736.00	0.06
	Largest	**4.71**	0.75	3.00	14.00	0.97
	Tree2	481.32	400.48	397.00	566.00	0.06
	Tree3	11.33	11.24	1.00	25.00	0.90
	Complete	39.25	37.75	15.00	66.00	0.19
$s = 0.02$	Edges	722.33	1,349.58	582.00	967.00	0.38
	Largest	**9.79**	20.27	4.00	56.00	0.97
	Tree2	497.56	407.99	416.00	581.00	0.03
	Tree3	16.38	16.05	3.00	34.00	0.72
	Complete	50.79	48.02	24.00	81.00	0.15
$s = 0.03$	Edges	1,090.00	16,537.54	808.00	2,360.00	0.97
	Largest	**40.15**	456.43	8.00	172.00	0.97
	Tree2	501.78	424.81	409.00	592.00	0.06
	Tree3	20.80	20.37	4.00	43.00	0.47
	Complete	61.55	58.15	33.00	93.00	0.14
$s = 0.04$	Edges	2,177.58	284,697.22	1,219.00	7,591.00	0.97
	Largest	**122.45**	2,833.45	18.00	354.00	0.97
	Tree2	492.44	425.42	412.00	578.00	0.01
	Tree3	22.28	21.94	6.00	44.00	0.46
	Complete	66.05	63.26	36.00	99.00	0.19

Table S3: Summary statistics of IBD graphs for different selection coefficients and the population bottleneck demographic scenario. There is directional selection with different selection coefficients $s \in [0.01, 0.02, 0.03, 0.4]$. The same description of IBD graph features as in Table 1. Shapiro-Wilk tests at the significance level 0.05 are performed with 250 replicates for 150 simulations, and the proportion of rejected null hypotheses reported as S.W.t. The sample size is five thousand diploid individuals. The Morgans length threshold is 0.03.
